# Effects of the selective TrkA agonist gambogic amide on pigmentation and growth of human hair follicles *in vitro*

**DOI:** 10.1371/journal.pone.0221757

**Published:** 2019-08-29

**Authors:** Remo Campiche, Maria Daniltchenko, Dominik Imfeld, Eva M. J. Peters

**Affiliations:** 1 DSM Nutritional Products, Personal Care & Aroma, Kaiseraugst, Switzerland; 2 Department of Psychosomatic Medicine, Charité Center (CC12) for Internal Medicine and Dermatology, Universitätsmedizin-Charité, Berlin, Germany; 3 Psychoneuroimmunology Laboratory, Department of Psychosomatic Medicine and Psychotherapy, University Hospital Giessen and Marburg, Giessen, Germany; Wenzhou Medical University, CHINA

## Abstract

The human hair follicle is a neuroendocrine mini-organ that can be used to study aging processes in vitro. Neurotrophins maintain homeostasis in hair biology via the Trk-family of receptors. TrkA, the high affinity receptor for nerve growth factor (NGF), is expressed in hair follicle melanocytes and keratinocytes, where it regulates proliferation, differentiation and apoptosis and may thereby play a role in hair pigmentation and growth. We investigated TrkA expression during the human hair cycle and the effects of a selective high affinity TrkA agonist, Gambogic Amide, on hair pigmentation and hair growth in human hair follicles in vitro. In human scalp skin, TrkA expression was strongest in proliferating melanocytes re-establishing the pigmentary unit in the hair bulb during the early hair growth phase, anagen. During high anagen and in the de-composing pigmentary-unit of the regression phase, catagen, bulb-melanocytes lost TrkA expression and only undifferentiated outer root sheath melanocytes maintained it. In cultured human anagen hair follicles, Gambogic Amide was able to prevent gradual pigment loss, while it stimulated hair shaft elongation. This was achieved by increased melanocyte activation, migration and dendricity, highlighted by distinct c-KIT-expression in melanocyte sub-populations. Our results suggest that Gambogic Amide can maintain hair follicle pigmentation by acting on undifferentiated melanocytes residing in the outer root sheath and making them migrate to establish the pigmentary-unit. This suggests that the selective TrkA agonist Gambogic Amide acts as an anti-hair greying and hair growth promoting molecule in vitro.

## Introduction

In today’s society healthy aging is crucial for well-being. One of the clinically visible signs of aging is greying hair [1]. This is why greying, the reversion of greying as well as the maintenance of hair pigmentation have been subject to extensive research in both academia and cosmetic industry. Past research efforts in this vein successfully employed the hair follicle as an exemplary mini-organ for the study of cellular and molecular processes of aging in general [2, 3]. Moreover, the hair follicle can be cultured and employed to study pharmacologic interventions [4]. However, hair follicle pigmentation and growth are complex processes and it is a challenge to find effective and safe means for anti-greying and hair growth applications to be used in clinical praxis.

Hair follicle pigmentation involves exact temporal and spatial regulation of the cells that produce the pigment, the melanocytes. Melanocyte turnover within the hair follicle, meaning renewal (recruitment, migration and proliferation), maturation (differentiation) and removal (senescence and apoptosis) is highly regulated to prevent melanoma development and spontaneously occurring primary hair follicle melanoma is in fact rarely reported [5]. To prevent and treat greying, interference with hair follicle melanocytes seems inevitable and thus the risk of melanoma induction looms.

To identify safe intervention strategies, it is therefore necessary to monitor melanocyte dynamics during hair growth. Hair shaft pigmentation, also called follicular melanogenesis, in both mice and humans is tightly linked to the growth phase (anagen) of the hair cycle [6–8], which is followed by a regression phase (catagen) and a resting phase (telogen) [9]. During early anagen, melanoblasts are recruited from the hair follicle bulge region in the outer root sheath and migrate to the hair follicle bulb where they start establishing the pigmentary-unit [9–11]. Melanocytes mature during high anagen and are then fully functional with respect to melanogenesis. When catagen is induced and the hair follicle starts to regress, the pigmentary-unit decomposes and melanocytes are removed from the regressing hair follicle by programmed cell death [9]. This melanocyte turnover during hair cycling can be monitored by various cellular markers such as Trp2 (early melanocyte differentiation), c-KIT (melanocyte migration), NKI-beteb (mature melanocytes), Ki67 (cell proliferation); p16 (maturation, senescence), or TUNEL-staining (apoptosis) demonstrating the tight regulation of proliferation, migration and finally death of melanocytes during the hair cycle.

Ideally, the mechanisms allowing hair pigmentation to be reestablished with each hair cycle are in a state of homeostasis. This homeostasis works optimally for the first 10–15 hair follicle growth cycles (until about 40 years of age). Thereafter, the mechanisms that maintain pigmentation seem to exhaust and the regeneration potential of the pigmentary-unit diminishes, leading to grey or white hair [12].

An agent stimulating hair follicle pigmentation should improve homeostasis but not interfere with it. One of the main routes studied to improve hair pigmentation focusses on melanin synthesis in hair follicle melanocytes and the gradual loss of factors maintaining it, such as alpha-melanocytes stimulating hormone (alpha-MSH) [13]. In addition, oxidative stress damage to hair follicle melanocytes is well established as one of the main causes of hair greying and involves declining anti-oxidant availability [14] and the lack of both genomic and mitochondrial DNA-repair [15, 16]. Further investigations into regulation of hair greying included comparative gene-expression analysis of pigmented, greying and white hair follicles [3, 17–19]. Our own such study revealed down-regulation of a set of genes involved in melanogenesis like tyrosinase, tyrosinase-related protein, Melan-A and SILV, and melanocyte migration like c-KIT but also regulators of oxidative stress like glutathione peroxidase and calpain 3 [3]. We could also show that greying is associated with non-optimal energy metabolism represented by dysregulated glutamine processing possibly leading to down-regulation of the neurotransmitter and signaling molecule glutamate, and up-regulation of GABA.

Skin has documented neuroendocrine properties [20, 21], and specifically melanogenesis is under hormonal regulation (nicely reviewed by Slominski et al 2004 [22]). It is now well established that also the hair follicle is a neuroendocrine organ and that hair growth and pigmentation are modulated by neurotrophins (NT) [13, 23, 24]. Hair follicle keratinocytes express functional receptors for various neurotrophins such as the high affinity Trk-family of receptors [23–25] and the low affinity p75NTR [26]. Previous studies suggest that the best studied neurotrophin, nerve growth factor (NGF) acts via TrkA to promote hair growth while its precursor pro-NGF acts via p75NTR to promote apoptosis and drive hair follicles into catagen [26]. Being derived from the neural crest, melanocytes are neuroendocrine cells [27, 28] that express NTs and their receptors [29, 30] and it was shown that NTs up-regulate the expression of Trp1 and tyrosinase in epidermal melanocytes [29]. Moreover, NGF and its high affinity receptor TrkA were found in hair follicle melanocytes during anagen.

The question therefor arises, if expression and manipulation of TrkA plays a role in hair follicle pigmentation homeostasis [31]. Gambogic Amide, a derivative of a natural product, gambogic acid and the major active ingredient in gamboge, a resin secreted by the *Garcinia hanburryi* tree in South-eastern Asia [32], was identified in a screen for small-molecule agonists to the TrkA receptor, selectively inducing its tyrosine phosphorylation. Furthermore, Gambogic Amide prevented neurons from glutamate-induced apoptosis and induced neurite outgrowth in PC12 cells [33]. In addition, it was found that Gambogic Amide is able to induce TrkA protein-expression [34]. Based on this knowledge we hypothesized that Gambogic Amide could be a promising molecule regulating melanocyte biology and pigmentation. Therefore, we investigated the effects of Gambogic Amide on melanocytes and pigmentation as well as growth of human hair follicle organ culture.

## Material and methods

### Tissue collection and donors

Tissue collection was essentially done as previously described [3]. In brief: Following Declaration of Helsinki principles and after obtaining approval from the institutional review board at Universitätsmedizin Charité Berlin, and written informed patient consent, temporal scalp skin was obtained from elective plastic surgery (face lifting) on healthy postmenopausal females between 40 and 75 years of age and processed as described before [4, 26, 35]. Samples had been obtained from plastic surgeons in collaboration since 2007. Full thickness skin biopsies were processed for histomorphometric assessment as described previously [3, 26, 36] and below. In addition, approximately 30 anagen VI HF per donor were sorted under an inverted microscope and further processed for immunohistochemistry and HF organ culture as described below.

### Human hair follicle organ culture

Anagen VI HF were cultured for seven days in supplemented Williams’ E medium (Biochrom AG, Berlin, Germany) as published before [15, 37]. On days 1, 4 and 7 medium and supplements were replaced, and pigmentation status and total length of each HF was documented. During this culture period, hair follicles maintain growth but gradually lose pigment similar to the loss of hair pigmentation during the greying process. HF were harvested on day 7 in Histogel embedding medium (Vector Laboratories, Peterborough, UK) for immunohistochemistry. Culture experiments were performed on at least three donor samples per group if not otherwise indicated in the figure legends. Hair follicles were treated with NGF (Boehringer Ingelheim, Ingelheim am Rhein, Germany) and Gambogic Amide (Gaia Chemical Corporation, Gaylordsville, CT, US) for seven days as indicated in figures. Controls were DMSO treated which was used as Gambogic Amide vehicle.

### Routine- and immunohistochemistry

8-μm-thick longitudinal cryosections through full thickness human scalp skin and cultured HF were processed and analyzed using a digital image analysis system (AxioVision, Zeiss, Göttingen, Germany) as previously described [3]. For NKI-beteb (detects Pmel17 [38]), c-KIT (receptor for SCF), TrkA; expressions were immunohistochemically detected following adapted established protocols (Table 1) [15, 37, 39]. Likewise, for hair cycle staging [39] and dystrophy screening Hematoxyline-Eosin (Merck, Darmstadt, Germany) staining was performed.

**Table 1:** Antibodies employed for Immunohistochemistry.

Antigen	Species	Company	Staining Method	Working Dilution
**NKI-beteb** **(Pmel17)**	mouse-anti-human	Monosan, Uden, Netherlands	IF (green) double with KIT (red)	1:20
**c-KIT**	rabbit-anti-human	Dako, Hamburg, Germany	IF (red) double with NKI-beteb (green)	1:100
**TrkA**	rabbit-anti-human	Santa Cruz Biotechnologie, Inc., Heidelberg, Germany	IF (red) double with NKI-beteb (green)	1:50
**p16**	rabbit-anti-human	Santa Cruz Biotechnologie, Inc., Heidelberg, Germany	IF (red) double with NKI-beteb (green)	1:50

For red label tetramethyl-rhodamine isothiocyanate-labeled, for green label Cy2-labeled goat anti-rabbit or goat anti-rat secondary antibodies (1:200; Jackson ImmunoResearch, West Grove, PA) were used. Secondary antibodies were added for 1 hour at 37°C in PBS with 2% normal goat serum. All sections were counterstained with 4-,6-diamidino-2-phenylindol-dihydrochlorid (DAPI; Boehringer Mannheim, Mannheim, Germany) for identification of cell nuclei. Abbreviations used: IF–immunofluorescence.

### Statistical evaluation

Statistical analysis was performed as previously described [3]. Means were calculated, and significant differences have been determined by Mann-Whitney U Test for unpaired samples. Significance was assumed if p*<0.05, p**<0.01 or p***<0.001 and is indicated accordingly in the figures.

## Results

### TrkA is expressed by undifferentiated melanocytes during the hair cycle

**Fig 1:**
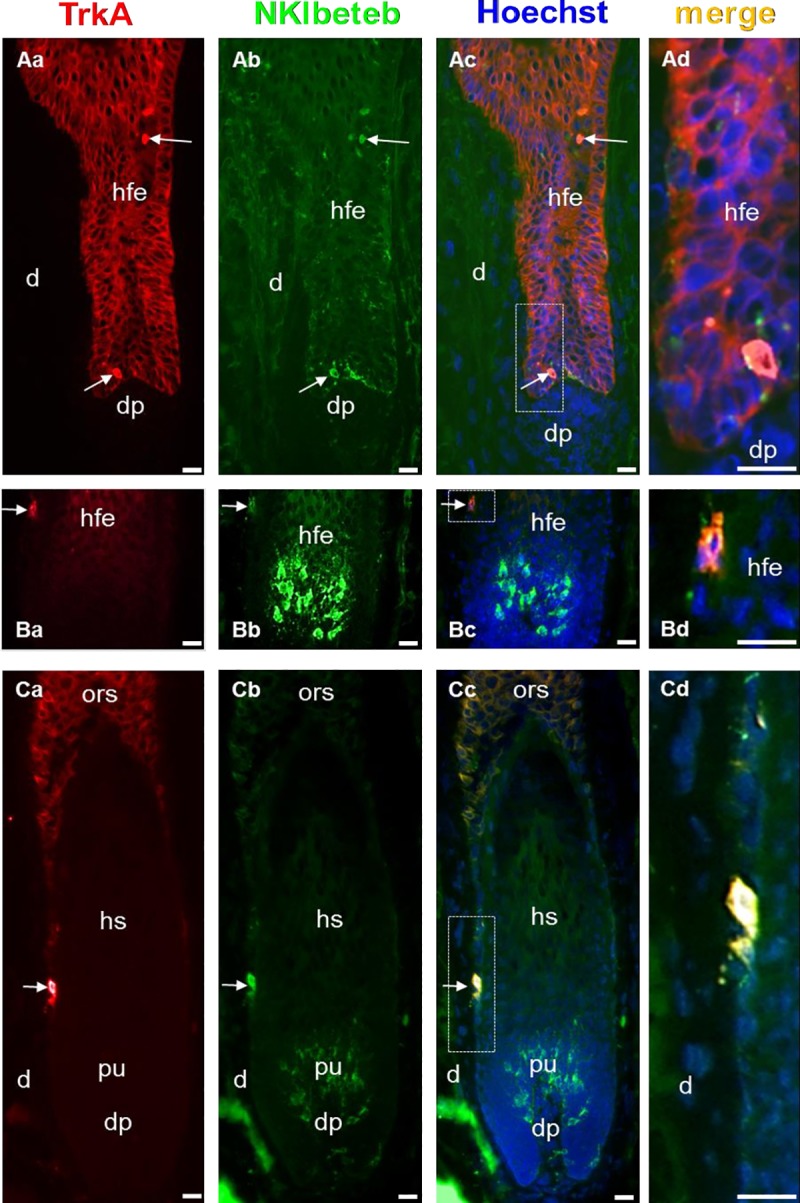
TrkA immunoreactivity in native human scalp skin hair follicles. a-TrkA, b-NKIbeteb, c-merge with Hoechst counterstaining of nuclei, d-magnified insert indicated by white box in c. **A: melanocytes re-establishing the pigmentary-unit during early anagen express TrkA.** Hair follicles contain dendritic TrkA- and round, undifferentiated TrkA+ cells (arrow) in the re-establishing pigmentary-unit. **B: melanocytes in the established pigmentary-unit during high anagen do not express TrkA or Ki67.** Hair follicles contain dendritic TrkA- (arrow) melanocytes in the hair bulb pigmentary-unit, while a round, undifferentiated TrkA+ melanocyte (arrow) is detectable in the outer root sheath of the hair bulb. **C: melanocytes in the decomposing pigmentary-unit during catagen do not express TrkA.** Hair follicles contain dendritic TrkA-melanocytes in the hair bulb pigmentary-unit (pu), while a round, undifferentiated TrkA+ melanocyte (arrow) is detectable in the outer root sheath (ors) of the hair bulb. Abbreviations: d–dermis, dp–dermal papilla, hfe–hair follicle epithelium, pu-pigmentary unit. Bars = 15 μm.

Native human scalp skin was investigated for TrkA expression of distinct sub-populations of hair follicle melanocytes during the hair cycle and showed a staining of keratinocytes in the outer root sheath throughout. In addition, in early anagen hair follicles round undifferentiated melanocytes in the re-establishing pigmentary-unit strongly expressed TrkA (Fig 1). In high anagen hair follicles with a fully establishment pigmentary-unit, melanocytes in the unit were all TrkA-negative while undifferentiated TrkA-positive melanocytes were found in the outer root sheath, which harbours the occasional melanoblast (Fig 1). In catagen the de-composing pigmentary-unit housed TrkA-negative melanocytes while, reminiscent to anagen, TrkA-positive melanocytes were found in the outer root sheath (Fig 1).

### Low NGF concentrations prevent pigment loss in cultured human hair follicles

**Fig 2:**
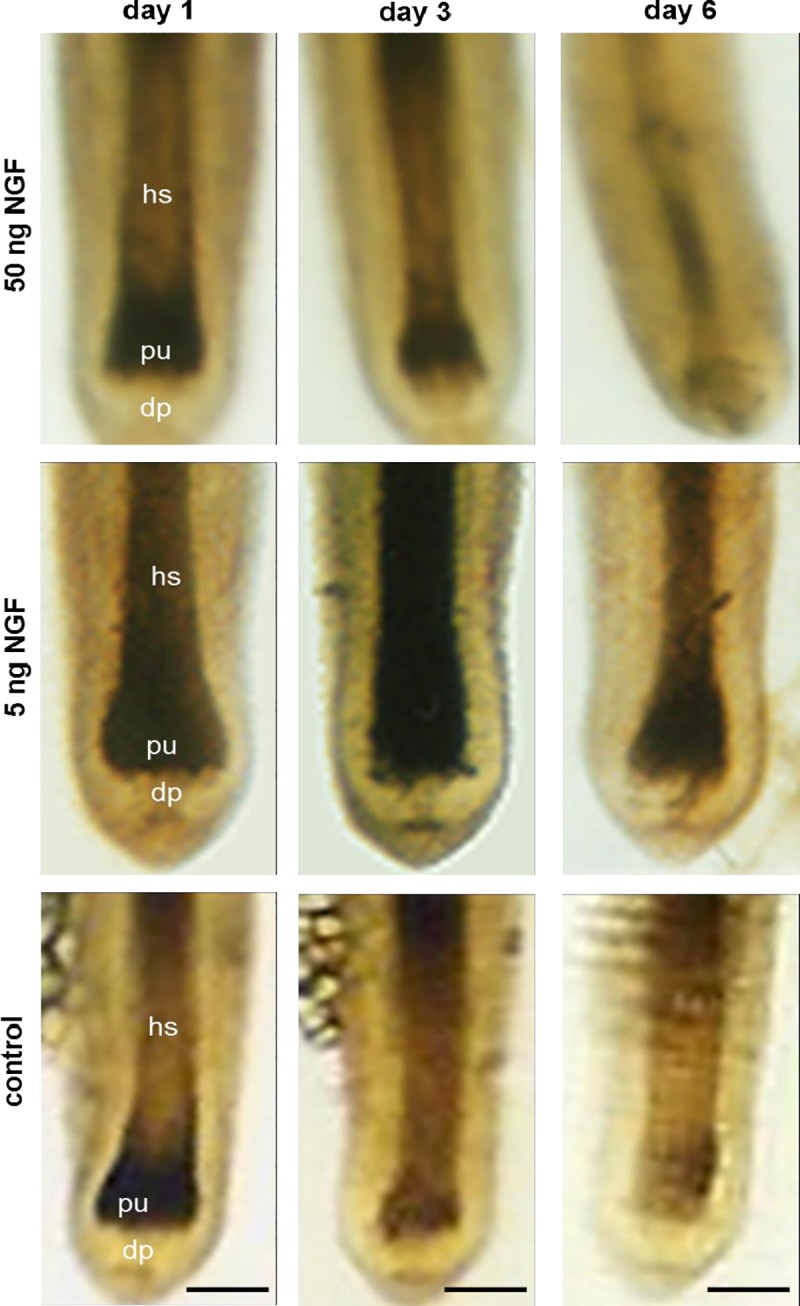
Effect of NGF on hair follicle pigmentation. Low concentration (5 ng/ml) of NGF maintained pigmentation of hair follicles in-vitro. However, high concentration (50 ng/ml) of NGF led to a rapid pigment loss similar as in control cultures. Bars = 50 μm.

Next the capacity of the TrkA agonist nerve growth factor (NGF) to modulate pigmentation in cultured human hair follicles was tested. 5 ng/ml NGF maintained pigmentation in cultured human anagen hair follicles (Fig 2) over the course of 7 days of incubation with NGF. Interestingly, this pro-pigmentary effect was absent when 50 ng/ml NGF were used (Fig 2), resembling the previously reported contrasting effects of 5 versus 50 ng/ml NGF on hair growth [26].

### High concentrations of Gambogic Amide repress pigment loss in cultured human hair follicles

**Fig 3:**
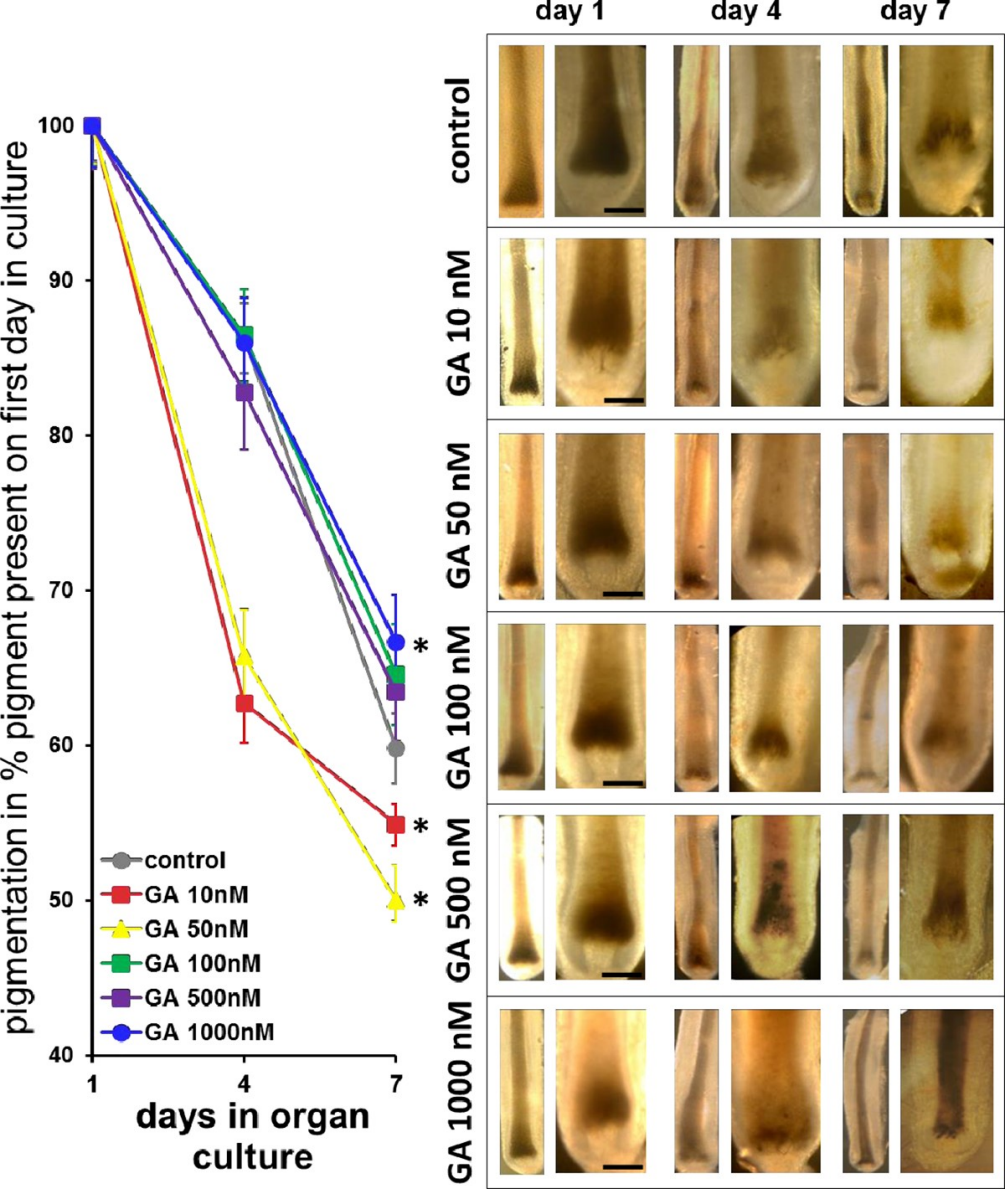
Macroscopic analysis of ongoing anagen VI hair follicle pigmentation in organ culture. Photomicrograph examples of one representative hair follicle per group are shown on the right. Please note maintenance of anagen VI morphology in the control hair follicle which is at the same time gradually loosing pigment. High concentrations of Gambogic Amide showed the capacity to maintain pigment. Bars = 50 μm.

Based on the findings in Figs 1 and 2 we chose to investigate the effect of Gambogic Amide, a selective TrkA agonist identified in a screen for small molecule TrkA agonists [33], on the pigmentation of cultured hair follicles. As depicted in Fig 3, Gambogic Amide at 100 nM and above was able to slow down the de-pigmentation process normally seen in cultured human anagen hair follicles significantly at doses of 100 to 1000 nM, and without signs of toxicity (Fig 3).

### Histomorphometry characterizes melanocyte biology during hair pigmentation homeostasis

**Fig 4:**
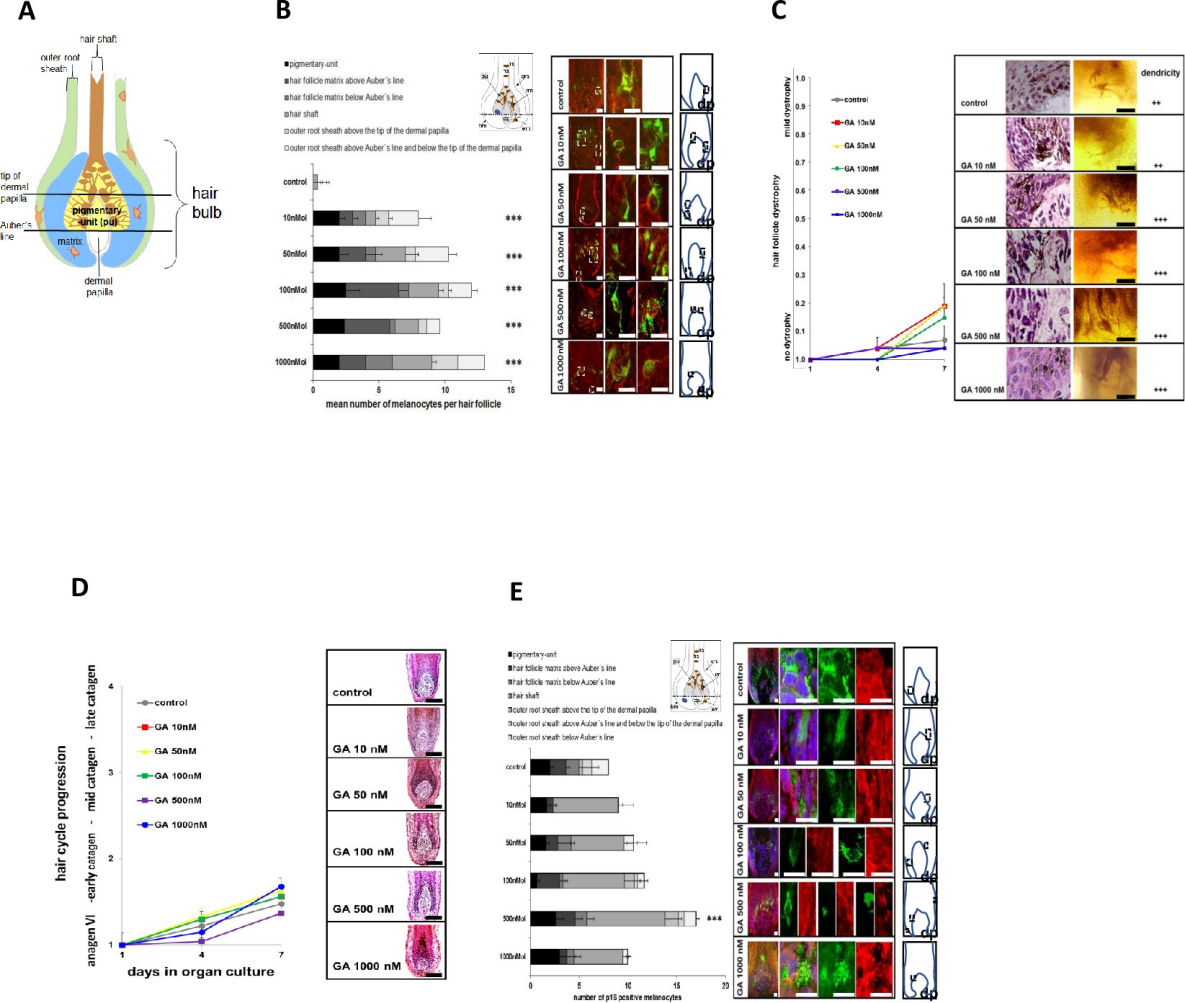
**A: Scheme of different hair follicle bulb compartments**. Highly dendritic melanocytes of the pigmentary-unit (yellow) are located above the Auber’s line in contact with the basement membrane. Outside the pigmentary-unit (blue) and in the outer root sheath (green) they do not serve hair pigmentation. In the outer root sheath melanocytes rest as oligo-dendritic cells. Abbreviations: PU—pigmentary-unit. **B: Immunohistochemical quantification of melanocytes with c-KIT, a marker for migrating and differentiating melanocytes**. Melanocytes are labelled green, c-KIT-immunoreactive cells are labelled red. White boxes indicate origin of high magnification blow-ups. Bars = 15 μm. **C: Macroscopic and microscopic analysis of dystrophy development and dendricity of melanocytes in the hair bulb of human hair follicles treated with Gambogic Amide.** Graph indicates degree of dystrophy. Photomicrograph examples of one representative hair follicle per group are shown on the right as derived from binocular microscopy of cultured hair follicles as well as hematoxylin&eosin routine staining. A blow-up image of the pigmentary-unit just above the dermal papilla is shown in each micrograph. Bars = 15 μm. **D: Macroscopic analysis of hair cycle progression in organ culture.** Photomicrograph examples of one representative hair follicle per group routinely stained with hematoxylin&eosin are shown on the right. Note the decreasing diameter of the hair follicle bulb and keratinocyte population in the hair follicle matrix on both sides of the dermal papilla with hair cycle progression in hair follicles treated with 1000 nM Gambogic Amide. Hair follicles treated with lower concentrations of Gambogic Amide kept their anagen-like appearance. Bars = 50 μm. **E: Immunohistochemical quantification of melanocytes with p16, a marker for mature and senescent melanocytes**. Melanocytes are labelled green, p16-immunoreactive cells are labelled red. White boxes indicate origin of high magnification blow-ups. Bars = 10 μm.

To analyze target melanocyte subpopulations of Gambogic Amide we investigated c-KIT-expression of hair follicle melanocytes in response to Gambogic Amide in various compartments of the hair bulb after 7 days of culture (Fig 4A). While control hair follicles only expressed a small number of c-KIT+ rounded up melanocytes in the hair shaft, there was an increase in c-KIT+ melanocytes in all hair follicle compartments studied in hair follicles treated with 10–1000 nM Gambogic Amide (Fig 4B) except for the outer root sheath below Auber’s line, where there were no c-KIT+ melanocytes found. The most dramatic increase in c-KIT+ melanocytes was found in the compartments above the Auber’s line (Fig 4B), suggesting enhanced activation and migration of differentiating melanocyte precursors.

Furthermore, we observed a slight increase in melanocyte dendricity in response to Gambogic Amide (50–1000 nM) (Fig 4C). No signs of dystrophy were observed during the entire course of treatment (Fig 4C).

To control, if these effects occurred within the anagen phase or were associated with premature catagen development in cultured anagen hair follicles, hair follicles exposed to Gambogic Amide were subjected to hair cycle progression analysis. By histomorphometry, Gambogic Amide at all tested concentrations did not significantly alter hair cycle progression compared to control samples (Fig 4D). In fact, over the course of 7 days exposure in vitro the hair follicles were still in anagen phase.

Concerning melanocyte turnover and cellular homeostasis, we found here an increased p16 expression at all tested concentrations with a peak at 500 nM of Gambogic Amide in melanocytes of all follicular compartments (Fig 4E). However, no particular increase in Trp2 and TUNEL expression was seen (not shown).

### Gambogic amide is also a hair growth promotor in cultured human anagen hair follicles

**Fig 5:**
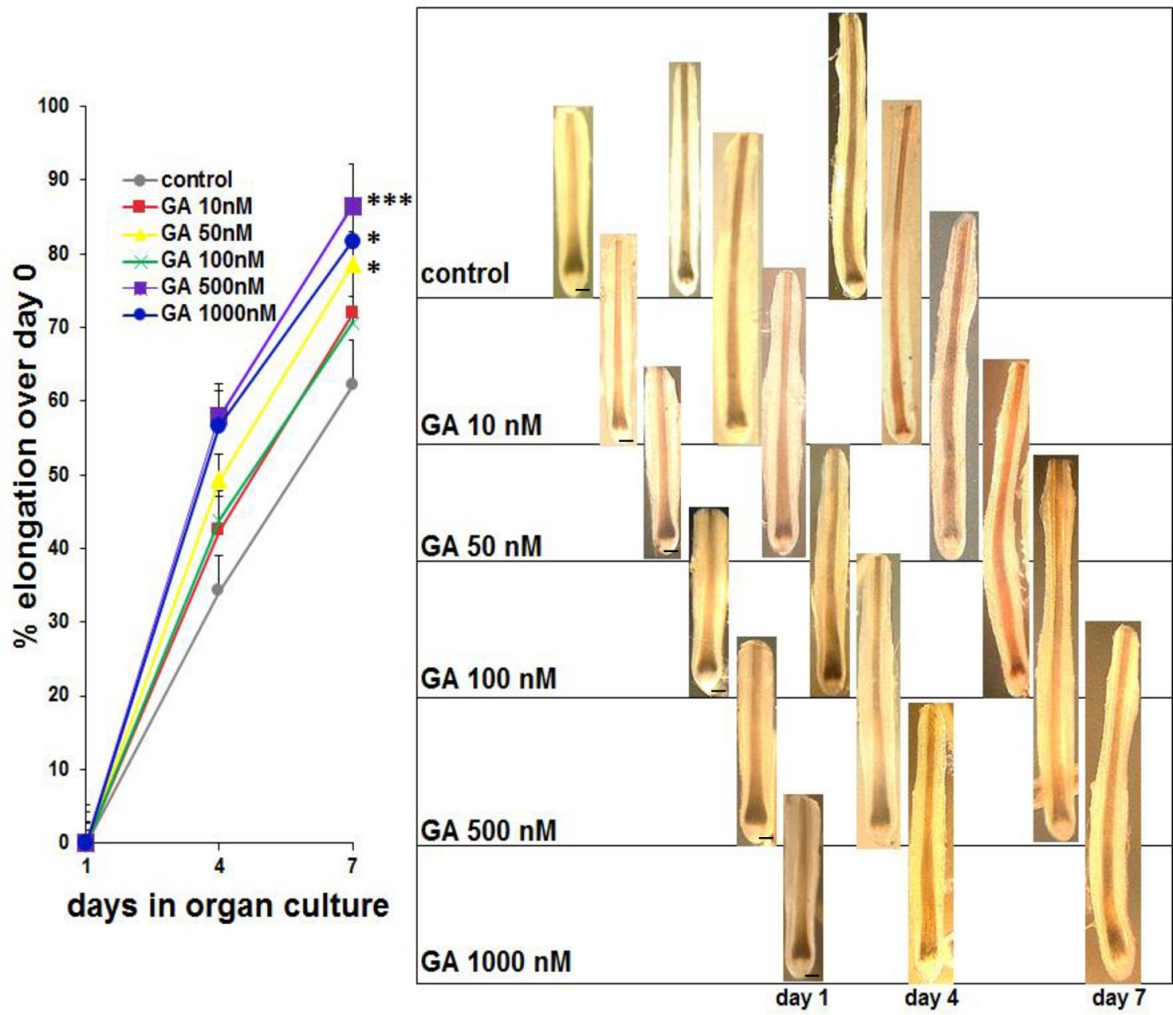
Macromorphic analysis of hair shaft elongation as a measure for hair growth in culture. Photomicrograph examples of one representative hair follicle per group are shown on the right of the statistical analysis. Here as well as for the generation of the following figures data has been pooled from three different donor samples. Each donor sample consisted of 3 wells containing 3 hair follicles each per tested concentration of GA comprising a total number of 27 hair follicles per group. Statistical analysis was performed by Mann-Withney-U Test for unpaired samples, p<0,05 = *, p<0,001 = ***. Standard errors are indicated. Bars = 50 μm.

Finally, growth of hair follicles exposed to Gambogic Amide was analyzed and revealed that in addition to hair pigmentation maintenance, Gambogic Amide was able to increase hair shaft elongation at all concentrations tested (Fig 5). This further underpinned the capability of Gambogic Amide to safely act as a pro-pigmenting agent in hair follicles without promotion of premature hair follicle regression or induction of hair dystrophy.

## Discussion

In this study we investigated the expression of TrkA in human hair follicles and studied the effects of a selective, plant derived TrkA agonist Gambogic Amide on the pigmentation of hair follicles in vitro to investigate its potential as an anti-aging ointment. We found TrkA expression during the human hair cycle in distinct melanocyte subpopulations suggestive of its expression primarily in undifferentiated melanocytes and potentially melanoblasts (Figs 1 and 2). Also, NGF and Gambogic Amide, both acting through the TrkA tyrosine kinase receptor, were able to slow-down the de-pigmentation process normally observed in vitro in cultured human anagen hair follicles and the histomorphometry of treated hair follicles suggested mobilization of un-differentiated melanocytes in the outer root sheath (Figs 3 and 4).

In the human scalp skin, we found that TrkA was strongly expressed in hair follicle melanocytes in the outer root sheath and in the developing pigmentary-unit at the beginning of anagen as well as in the outer root sheath of fully developed anagen hair follicles. This is in line with previous findings showing that TrkA is mainly expressed in melanocytes and keratinocytes in anagen hair follicles [31]. However, differentiated melanocytes in the fully developed pigmentary-unit of high anagen hair follicles in the analysis presented here were TrkA-negative. Hence, melanocytes presumably lost TrkA expression sometime during the establishment of the pigmentary-unit from early into high anagen. TrkA agonists such as NGF and Gambogic Amide can therefore mainly act on undifferentiated TrkA-positive melanocytes or melanoblasts, which are cells with high proliferative and migratory potential when activated.

In addition, TrkA-positive melanocytes were round-shaped and expressed the melanocyte migration marker c-KIT. In mice, c-KIT was previously shown to be predominantly expressed in migrating melanoblasts in the developing hair follicle [40] and it is known to be a survival marker for migrating and differentiating melanocytes [41] required for the cyclic regeneration of the pigmentary unit and hence pigmentation of hair follicles [42]. Indeed, it was shown in mice that treatment with [4-t-Butylphenyl]-N-(4-imidazol-1-yl phenyl)sulfonamide (ISCK03) an inhibitor of c-KIT signaling abolished melanin synthesis leading to depigmentation of newly grown hair [43]. In humans, it is well established that mutations in the c-KIT receptor gene lead to piebaldism which is characterized by patchy depigmentation of body, scalp and hair [44, 45]. We here found that Gambogic Amide significantly induced the expression of c-KIT in melanocytes in the outer root sheath as well as in hair follicle compartments above the Auber’s line where they contribute to hair follicle pigmentation.

In line with c-KIT being also a melanocyte differentiation marker, the melanocytes in hair follicle treated with Gambogic Amide also displayed slightly enhanced dendricity. Since melanocytes are derived from the neural crest it resembled the capability of Gambogic Amide to induce neurite outgrowth in various neuronal cell lines [33, 34]. In addition, induction of the NGF receptor in epidermal melanocytes with phorbol 12-tetradecanoate 13-acetate (TPA) correlated with the appearance of a more dendritic morphology [46].

Looking at hair follicle pigmentation we found that Gambogic Amide showed a similar activity as nerve growth factor NGF, which is the natural high affinity agonist of TrkA. In contrast to NGF however, we found that Gambogic Amide showed pro-pigmenting activity dose-dependently at higher concentrations, whereas NGF showed a maximum at 5 ng/ml. High concentrations of NGF like 50 ng/ml decreased hair follicle pigmentation in vitro (Fig 2). Notably, the NGF preparation used in this study is the same as was used previously by Peters et al. [26]. It contains low amounts of pro-NGF and hence also acts on p75NTR, the high affinity receptor for pro-NGF and the low affinity receptor for NGF. It may be that at a certain amount of pro-NGF activity on p75NTR outperforms the activity on TrkA and drives the hair follicles not only into catagen [26] but also into depigmentation. Interestingly, others found that NGF and Gambogic Amide displayed different kinetics in terms of activation of TrkA. NGF provoked initial activation by enhanced expression and fast degradation of TrkA whereas Gambogic Amide showed a more sustained TrkA activation pattern with a constantly increasing expression over three days in a human leukemia cell line [34]. A similar mechanism could be present in human hair follicles. Hence, we see better maintenance of in vitro hair follicle pigmentation in a dose-dependent manner with Gambogic Amide (Fig 3), rather than an optimum as with NGF (Fig 2). These conclusions are supported by the observation that a high dose of NGF failed to prevent pigmentation loss. NGF solutions obtained from in vivo sources contain both the active compound and the precursor proNGF, which has a high affinity to p75NTR. In contrast to NGF-TrkA interaction, proNGF-p75NTR interaction can induce apoptosis, especially when high proNGF is present and TrkA is low [47]. Increased levels of proNGF alongside NGF may also explain why NGF, released in high dosages during stress, shows conflicting results in studies addressing distress disorders [48–50]. In vivo, high dosage NGF effects may therefore be outweight by proNGF effects and specific TrkA agonists address desired TrkA activation more effectively.

We used here the Philpott model of hair follicles which are truncated below the bulge region. This means Gambogic Amide acted on undifferentiated melanoblasts released from the bulge already, and as such was able to sustain pigmentation in vitro more efficiently (Fig 3) without acting on stem cells. Thus, we propose that over-consumption of stem cells and subsequent pre-mature greying will no be an issue in vivo. However, we cannot completely exclude that there could be a feedback loop in full length hair follicles leading to the over-consumption, or that Gambogic Amide exerts other effects on the stem cells residing in the bulge.

In summary, we provide evidence that it is possible to halt the hair aging process represented by hair greying in vitro with Gambogic amide. An important mechanism how Gambogic Amide stimulates pigmentation in hair follicle melanocytes is via induction of c-KIT expression (Fig 4). Gambogic Amide seems to rebalance defects in c-KIT signaling occurring in human hair follicles in vitro leading to depigmentation simulating greying under normal culture conditions. As mentioned, hair follicle pigmentation is a tightly regulated process involving not only recruitment of melanocytes and establishment of a functional pigmentary unit, but also constant cell turnover by maturation, senescence and apoptosis. We found increased p16 expression in melanocytes of all follicular compartments, similar to the increase in c-KIT expression (Fig 4E). This underpins the observation that Gambogic Amide acts during a window of melanocyte migration, establishment of the pigmentary unit until the mature cells start senescence. This increase of senescent cell population together with an increase in melanogenic activity ensures follicular melanocyte homeostasis and aids to the notion of Gambogic Amide being safe as a potential anti-hair aging molecule.

The potential of Gambogic Amide or other molecules activating TrkA signaling in human hair follicle as a remedy to reverse signs of aging hair like greying is further corroborated by the finding that Gambogic Amide is able to induce hair follicle growth in vitro (Fig 5). In addition, we found that Gambogic Amide has no significant influence on the hair growth cycle since hair follicles treated with Gambogic Amide were found to be in the same growth phase (anagen) as control hair follicles (Fig 4D).

The notion that Gambogic Amide recruits melanocytes of the outer root sheath while the hair continues or even improves its growth activity is evidenced by the maintenance of anagen morphology and increased hair shaft elongation. This is intriguing, as it was shown previously that without treatment, in vitro pigmentation correlated negatively with hair shaft elongation in cultured human anagen hair follicles and vice-versa and that this links with up-regulated gene-expression of keratins involved in active hair growth [15, 51]. Our finding further underpinned the capability of Gambogic Amide to safely act as a pro-pigmenting agent in hair follicles.

In conclusion, we provide evidence that the TrkA agonist Gambogic Amide is a promising molecule able to protect hair follicle pigmentation in vitro by mobilizing TrkA and c-KIT-positive hair follicle melanocytes. Furthermore, Gambogic Amide has a growth promoting activity on hair follicles in vitro, further highlighting its potential as a hair health or hair anti-aging active.

## References

[1] Peters EM, Imfeld D, Gräub R. Graying of the human hair follicle. J Cosmet Sci. 2011;62:121–5. 21635841

[2] Paus R. Frontiers in the (neuro-)endocrine controls of hair growth. J Investig Dermatol Symp Proc. 2007;12(2):20–2. 10.1038/sj.jidsymp.5650050 . 18004292

[3] Peters EM, Liezmann C, Spatz K, Ungethum U, Kuban RJ, Daniltchenko M, et al Profiling mRNA of the graying human hair follicle constitutes a promising state-of-the-art tool to assess its aging: an exemplary report. J Invest Dermatol. 2013;133(5):1150–60. 10.1038/jid.2012.462 . 23235529

[4] Peters EM, Liotiri S, Bodo E, Hagen E, Biro T, Arck PC, et al Probing the effects of stress mediators on the human hair follicle: substance P holds central position. Am J Pathol. 2007;171(6):1872–86. 10.2353/ajpath.2007.061206 18055548 PMC2111110

[5] Tjarks BJ, Somani N, Piliang M, Bergfeld WF. A proposed classification for follicular involvement by melanoma. J Cutan Pathol. 2017;44(1):45–52. 10.1111/cup.12851 . 27778368

[6] Paus R, Foitzik K. In search of the ‘‘hair cycle clock”: a guided tour. Differentiation. 2004;72:489–511. 10.1111/j.1432-0436.2004.07209004.x 15617561

[7] Slominski A, Paus R. Melanogenesis is coupled to murine anagen: toward new concepts for the role of melanocytes and the regulation of melanogenesis in hair growth. J Invest Dermatol. 1993;101(1 Suppl):90S–7S. 10.1111/1523-1747.ep12362991 . 8326158

[8] Slominski A, Wortsman J, Plonka PM, Schallreuter KU, Paus R, Tobin DJ. Hair follicle pigmentation. J Invest Dermatol. 2005;124(1):13–21. 10.1111/j.0022-202X.2004.23528.x 15654948 PMC1201498

[9] Tobin DJ, Paus R. Graying: gerontobiology of the hair follicle pigmentary unit. Exp Gerontol. 2001;36:29–54. 11162910 10.1016/s0531-5565(00)00210-2

[10] Commo S, Bernard BA. Melanocyte subpopulation turnover during the human hair cycle: an immunohistochemical study. Pigment Cell Res. 2000;13(4):253–9. . 10952393 10.1034/j.1600-0749.2000.130407.x

[11] Commo S, Gaillard O, Bernard BA. Human hair greying is linked to a specific depletion of hair follicle melanocytes affecting both the bulb and the outer root sheath. Br J Dermatol. 2004;150(3):435–43. 10.1046/j.1365-2133.2004.05787.x . 15030325

[12] Tobin DJ. The aging hair follicle pigmentary unit. Household and Personal Care TODAY. 2007;(1):15–8.

[13] Paus R. A neuroendocrinological perspective on human hair follicle pigmentation. Pigment Cell Melanoma Res. 2011;24(1):89–106. 10.1111/j.1755-148X.2010.00808.x . 21108769

[14] Wood JM, Decker H, Hartmann H, Chavan B, Rokos H, Spencer JD, et al Senile hair graying: H2O2-mediated oxidative stress affects human hair color by blunting methionine sulfoxide repair. FASEB J. 2009;23(7):2065–75. 10.1096/fj.08-125435 . 19237503

[15] Arck PC, Overall R, Spatz K, Liezman C, Handjiski B, Klapp BF, et al Towards a "free radical theory of graying": melanocyte apoptosis in the aging human hair follicle is an indicator of oxidative stress induced tissue damage. FASEB J. 2006;20(9):1567–9. 10.1096/fj.05-4039fje . 16723385

[16] Seiberg M. Age-induced hair greying—the multiple effects of oxidative stress. Int J Cosmet Sci. 2013;35(6):532–8. 10.1111/ics.12090 . 24033376

[17] Matama T, Gomes AC, Cavaco-Paulo A. Hair Coloration by Gene Regulation: Fact or Fiction? Trends Biotechnol. 2015;33(12):707–11. 10.1016/j.tibtech.2015.10.001 . 26549772

[18] Yin L, Coelho SG, Valencia JC, Ebsen D, Mahns A, Smuda C, et al Identification of Genes Expressed in Hyperpigmented Skin Using Meta-Analysis of Microarray Data Sets. J Invest Dermatol. 2015;135(10):2455–63. 10.1038/jid.2015.179 25950827 PMC4567955

[19] Shi Y, Luo LF, Liu XM, Zhou Q, Xu SZ, Lei TC. Premature graying as a consequence of compromised antioxidant activity in hair bulb melanocytes and their precursors. PLoS One. 2014;9(4):e93589 10.1371/journal.pone.0093589 24695442 PMC3973559

[20] Slominski A, Wortsman J. Neuroendocrinology of the skin. Endocr Rev. 2000;21(5):457–87. 10.1210/edrv.21.5.0410 . 11041445

[21] Slominski AT, Zmijewski MA, Zbytek B, Tobin DJ, Theoharides TC, Rivier J. Key role of CRF in the skin stress response system. Endocr Rev. 2013;34(6):827–84. 10.1210/er.2012-1092 23939821 PMC3857130

[22] Slominski A, Tobin DJ, Shibahara S, Wortsman J. Melanin pigmentation in mammalian skin and its hormonal regulation. Physiol Rev. 2004;84(4):1155–228. 10.1152/physrev.00044.2003 . 15383650

[23] Botchkarev VA, Botchkareva NV, Peters EMJ, Paus R. Epithelial growth control by neurotrophins: leads and lessons from the hair follicle. 2004;146:493–513. 10.1016/s0079-6123(03)46031-7 14699982

[24] Botchkarev VA, Botchkarev NV, Albers KM, van der Veen C, Lewin GR, Paus R. Neurotrophin-3 involvement in the regulation of hair follicle morphogenesis. J Invest Dermatol. 1998;111(2):279–85. 10.1046/j.1523-1747.1998.00277.x . 9699730

[25] Botchkarev VA, Yaar M, Peters EM, Raychaudhuri SP, Botchkareva NV, Marconi A, et al Neurotrophins in skin biology and pathology. J Invest Dermatol. 2006;126(8):1719–27. 10.1038/sj.jid.5700270 . 16845411

[26] Peters EM, Stieglitz MG, Liezman C, Overall RW, Nakamura M, Hagen E, et al p75 Neurotrophin Receptor-Mediated Signaling Promotes Human Hair Follicle Regression (Catagen). Am J Pathol. 2006;168(1):221–34. 10.2353/ajpath.2006.050163 16400025 PMC1592649

[27] Slominski A, Paus R, Schadendorf D. Melanocytes as "sensory" and regulatory cells in the epidermis. J Theor Biol. 1993;164(1):103–20. 10.1006/jtbi.1993.1142 . 8264240

[28] Slominski A. Neuroendocrine activity of the melanocyte. Exp Dermatol. 2009;18(9):760–3. 10.1111/j.1600-0625.2009.00892.x 19558501 PMC2773661

[29] Marconi A, Panza MC, Bonnet-Duquennoy M, Lazou K, Kurfurst R, Truzzi F, et al Expression and function of neurotrophins and their receptors in human melanocytes. Int J Cosmet Sci. 2006;28:255–61. 10.1111/j.1467-2494.2006.00321.x 18489265

[30] Yaar M, Eller MS, DiBenedetto P, Reenstra WR, Zhai S, McQuaid T, et al The trk Family of Receptors Mediates Nerve Growth Factor and Neurotrophin-3 Effects in Melanocytes. J Clin Invest. 1994;94:1550–62. 10.1172/JCI117496 7929831 PMC295306

[31] Adly MA, Assaf HA, Nada EA, Soliman M, Hussein M. Expression of nerve growth factor and its high-affinity receptor, tyrosine kinase A proteins, in the human scalp skin. J Cutan Pathol. 2006;33(8):559–68. 10.1111/j.1600-0560.2006.00443.x . 16919030

[32] Obianyo O, Ye K. Novel small molecule activators of the Trk family of receptor tyrosine kinases. Biochim Biophys Acta. 2013;1834(10):2213–8. 10.1016/j.bbapap.2012.08.021 22982231 PMC3602283

[33] Jang SW, Okada M, Sayeed I, Xiao G, Stein D, Jin P, et al Gambogic amide, a selective agonist for TrkA receptor that possesses robust neurotrophic activity, prevents neuronal cell death. Proc Natl Acad Sci U S A. 2007;104(41):16329–34. 10.1073/pnas.0706662104 17911251 PMC2042206

[34] Shen J, Yu Q. Gambogic amide selectively upregulates TrkA expression and triggers its activation. Pharmacol Rep. 2015;67(2):217–23. 10.1016/j.pharep.2014.09.002 . 25712642

[35] Philpott MP, Green MR, Kealey T. Human hair growth in vitro. J Cell Sci. 1990;97:463–71. 1705941 10.1242/jcs.97.3.463

[36] Arck PC, Handjiski B, Peters EM, Hagen E, Klapp BF, Paus R. Topical minoxidil counteracts stress-induced hair growth inhibition in mice. Experimental dermatology. 2003;12(5):580–90. Epub 2004/01/07. . 14705798 10.1034/j.1600-0625.2003.00028.x

[37] Peters EM, Hansen MG, Overall RW, Nakamura M, Pertile P, Klapp BF, et al Control of human hair growth by neurotrophins: brain-derived neurotrophic factor inhibits hair shaft elongation, induces catagen, and stimulates follicular transforming growth factor beta2 expression. J Invest Dermatol. 2005;124(4):675–85. 10.1111/j.0022-202X.2005.23648.x . 15816823

[38] Adema GJ, Bakker ABH, De Boer AJ, Hohenstein P, Figdor CG. pMel17 is recognised by monoclonal antibodies NKI-beteb, HMB-45 and HMB-50 and by anti-melanoma CTL. Brit J Cancer. 1996;73:1044–8. 10.1038/bjc.1996.202 8624261 PMC2074403

[39] Muller-Rover S, Handjiski B, van der Veen C, Eichmuller S, Foitzik K, McKay IA, et al A comprehensive guide for the accurate classification of murine hair follicles in distinct hair cycle stages. J Invest Dermatol. 2001;117(1):3–15. 10.1046/j.0022-202x.2001.01377.x . 11442744

[40] Peters EMJ, Tobin DJ, Botchkareva N, Maurer M, Paus R. Migration of Melanoblasts into the Developing Murine Hair Follicle Is Accompanied by Transient c-Kit Expression. J Histochem Cytochem. 2002;50(6):751–66. 10.1177/002215540205000602 12019292

[41] Alexeev V, Yoon K. Distinctive role of the cKit receptor tyrosine kinase signaling in mammalian melanocytes. J Invest Dermatol. 2006;126(5):1102–10. 10.1038/sj.jid.5700125 . 16410786

[42] Botchkareva NV, Khlgatian M, Longley BJ, Botchkarev VA, Gilchrest BA. SCF/c-kit signaling is required for cyclic regeneration of the hair pigmentation unit. FASEB J. 2001;15:645–58. 10.1096/fj.00-0368com 11259383

[43] Na YJ, Baek HS, Ahn SM, Shin HJ, Chang IS, Hwang JS. [4-t-butylphenyl]-N-(4-imidazol-1-yl phenyl)sulfonamide (ISCK03) inhibits SCF/c-kit signaling in 501mel human melanoma cells and abolishes melanin production in mice and brownish guinea pigs. Biochem Pharmacol. 2007;74(5):780–6. 10.1016/j.bcp.2007.05.028 . 17658483

[44] Syrris P, Heathcote K, Carrozzo R, Devriendt K, Elcioglu N, Garrett C, et al Human piebaldism: six novel mutations of the proto-oncogene KIT. Hum Mutat. 2002;20(3):234 10.1002/humu.9057 . 12204004

[45] Boissy RE, Nordlund JJ. Molecular Basis of Congenital Hypopigmentary Disorders in Humans: A Review. Pigment Cell Res. 1997;10:12–24. 9170158 10.1111/j.1600-0749.1997.tb00461.x

[46] Peacocke M, Yaar M, Mansur CP, Chao MV, Gilchrest BA. Induction of nerve growth factor receptors on cultured human melanocytes. PNAS. 1988;85:5282–6. 10.1073/pnas.85.14.5282 2839841 PMC281734

[47] Ioannou MS, Fahnestock M. ProNGF, but Not NGF, Switches from Neurotrophic to Apoptotic Activity in Response to Reductions in TrkA Receptor Levels. Int J Mol Sci. 2017;18(3). 10.3390/ijms18030599 28282920 PMC5372615

[48] Costa RO, Perestrelo T, Almeida RD. PROneurotrophins and CONSequences. Mol Neurobiol. 2018;55(4):2934–51. 10.1007/s12035-017-0505-7 . 28456935

[49] Caserta M, Ben-Soussan TD, Vetriani V, Venditti S, Verdone L. Influence of Quadrato Motor Training on Salivary proNGF and proBDNF. Front Neurosci. 2019;13:58 10.3389/fnins.2019.00058 30792622 PMC6374314

[50] Mondal AC, Fatima M. Direct and indirect evidences of BDNF and NGF as key modulators in depression: role of antidepressants treatment. Int J Neurosci. 2019;129(3):283–96. 10.1080/00207454.2018.1527328 . 30235967

[51] Choi HI, Choi GI, Kim EK, Choi YJ, Sohn KC, Lee Y, et al Hair greying is associated with active hair growth. Br J Dermatol. 2011;165(6):1183–9. 10.1111/j.1365-2133.2011.10625.x . 21916889

